# *catena*-Poly[copper(I)-bis(μ-trifluoro­methane­sulfonato-κ^2^ *O*:*O*′)-copper(I)-bis(μ-trimethyl trithio­phosphite)-κ^2^ *P*:*S*;κ^2^ *S*:*P*]

**DOI:** 10.1107/S1600536808041809

**Published:** 2008-12-17

**Authors:** Christoph E. Strasser, Stephanie Cronje, Helgard G. Raubenheimer

**Affiliations:** a Department of Chemistry and Polymer Science, University of Stellenbosch, Private Bag X1, Matieland, 7602, South Africa

## Abstract

The title compound, [Cu_2_(CF_3_SO_3_)_2_(C_3_H_9_PS_3_)_2_]_*n*_, crystallizes as infinite chains in which two trimethyl trithio­phosphite ligands and two trifluoro­methane­sulfonate anions bridge the essentially tetra­hedrally coordinated Cu^I^ ions in an alternating fashion. The P and one S atom of each trimethyl trithio­phosphite ligand are employed for coordination. The mol­ecular structure exhibits the rare motif of copper(I) bridged by two trifluoro­methane­sulfonate anions generating eight-membered rings.

## Related literature

For related structures, see: Blue *et al.* (2006 ▶); Kataeva *et al.* (1995 ▶, 2000 ▶); Knight & Keller (2006 ▶); Kursheva *et al.* (2003 ▶); Stibrany & Potenza (2007 ▶); Stibrany *et al.* (2006 ▶).
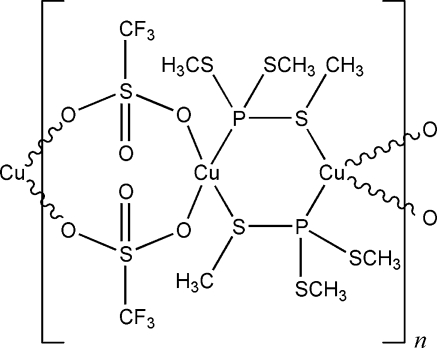


## Experimental

### 

#### Crystal data

[Cu_2_(CF_3_SO_3_)_2_(C_3_H_9_PS_3_)_2_]
*M* *_r_* = 769.78
Monoclinic, 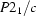

*a* = 8.8347 (15) Å
*b* = 18.306 (3) Å
*c* = 8.1731 (14) Å
β = 102.674 (3)°
*V* = 1289.6 (4) Å^3^
*Z* = 2
Mo *K*α radiation
μ = 2.49 mm^−1^
*T* = 100 (2) K
0.10 × 0.08 × 0.04 mm

#### Data collection

Bruker APEX CCD area-detector diffractometer
Absorption correction: multi-scan (*SADABS*; Bruker, 2002 ▶) *T*_min_ = 0.789, *T*_max_ = 0.907
7349 measured reflections
2612 independent reflections
2425 reflections with *I* > 2σ(*I*)
*R*_int_ = 0.021

#### Refinement

*R*[*F* ^2^ > 2σ(*F* ^2^)] = 0.030
*wR*(*F* ^2^) = 0.075
*S* = 1.04
2612 reflections
148 parameters
H-atom parameters constrained
Δρ_max_ = 0.87 e Å^−3^
Δρ_min_ = −0.42 e Å^−3^

### 

Data collection: *SMART* (Bruker, 2002 ▶); cell refinement: *SAINT* (Bruker, 2003 ▶); data reduction: *SAINT*; program(s) used to solve structure: *SHELXS97* (Sheldrick, 2008 ▶); program(s) used to refine structure: *SHELXL97* (Sheldrick, 2008 ▶); molecular graphics: *X-SEED* (Barbour, 2001 ▶); software used to prepare material for publication: *X-SEED*.

## Supplementary Material

Crystal structure: contains datablocks I, global. DOI: 10.1107/S1600536808041809/lh2742sup1.cif

Structure factors: contains datablocks I. DOI: 10.1107/S1600536808041809/lh2742Isup2.hkl

Additional supplementary materials:  crystallographic information; 3D view; checkCIF report

## Figures and Tables

**Table:** 

Cu1—S1^i^	2.2943 (8)
Cu1—P1	2.1895 (7)
Cu1—O1	2.1466 (18)
Cu1—O2^ii^	2.065 (2)

**Table:** 

P1—Cu1—S1^i^	121.88 (3)
O1—Cu1—S1^i^	105.35 (5)
O1—Cu1—P1	104.59 (6)
O2^ii^—Cu1—S1^i^	102.03 (7)
O2^ii^—Cu1—P1	123.32 (7)
O2^ii^—Cu1—O1	95.18 (8)

Symmetry codes: (i) 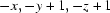; (ii) 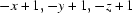.

**Fig. 1.**
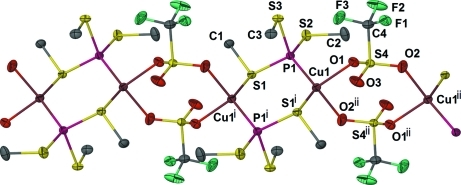
Part of an infinite chain formed by (I), unlabeled atoms are related by one unit cell translation along the a axis; ellipsoids are drawn at the 50% probability level. Symmetry operators: (i) = -x, -y + 1, -z + 1; (ii) = -x + 1, -y + 1, -z + 1.

## supplementary crystallographic information

### Comment

The structure of the title compound (I), consists of chains of tetrahedrally coordinated Cu^I^ centres that are bridged by two P(SMe)_3_ ligands in a κ^2^*P*:*S*-fashion, forming 6-membered rings in the chair conformation. Two trifluoromethanesulfonate anions each employ two oxygen atoms to bridge two copper atoms, yielding 8-membered rings. The copper atoms thus form spiro-junctions between the alternating 6- and 8-membered rings generated by the trithiophosphite and trifluoromethanesulfonate ligands; a point of symmetry is located in the centre of each ring.

The crystal structure of (I) therefore is related to compounds of the [CuX{P(S*R*)_3_}]_n_ (*R* = alkyl, phenyl; *X* = Cl, Br, I, SCN) type which also show the same arrangement of 6-membered rings formed by two trithiophosphite ligands where the copper centres also form spiro-junctions to the 4-membered (8-membered) rings generated by two bridging (pseudo)halide (thiocyanate) anions (Kataeva *et al.*, 1995; Kataeva *et al.*, 2000). Cu—P and Cu—S bond lengths in (I) are shorter than in most halide analogues. This difference might be caused by the harder and more electron-withdrawing nature of the trifluoromethanesulfonate counter-anion. The average Cu—P bond length in the halide complexes is 2.22 Å and the average Cu—S distance 2.39 Å. An exception is the cluster formed by triisopropyltrithiophosphite with 4 CuCl units [Cu—P 2.207 (7) Å, Cu—S 2.185 (8) Å] that also might exert a similar electron-withdrawing effect on the ligand (Kursheva *et al.*, 2003).

Tetrahydrofuran (thf), from which (I) was crystallized, does not act as a ligand towards Cu^I^ in the present structure. This behaviour of (I) is in contrast to the complex [Cu(CH_3_CN)_2_(PPh_3_)_2_]CF_3_SO_3_ which spontaneously yields crystals of [Cu(CF_3_SO_3_)(PPh_3_)_2_(thf)] when dissolved in thf (Knight & Keller, 2006). In the latter complex, the Cu—O(thf) bond is shorter [2.125 (2) Å] than the Cu—O(CF_3_SO_3_) bond [2.168 (2) Å] in (I).

Two copper centres bridged by two trifluoromethanesulfonate anions, each employing two oxygen atoms for coordination, is a very rare motif. Only two other structures of Cu^I^ (Stibrany *et al.*, 2006; Stibrany & Potenza, 2007) and one of Cu^II^ (Blue *et al.*, 2006) are known that exhibit such an arrangement. The former examples comprise tetrahedral Cu^I^ centres with the remaining sites occupied by phosphine or imine donors where the Cu—O distances of the former structure [2.111 (4) and 2.189 (4) Å] are comparable to those in (I) whereas such bonds are longer [2.336 (6) and 2.350 (7) Å] in the latter structure.

### Experimental

trimethyl trithiophosphite (202 mg, 1.2 mmol) was dissolved in thf (20 ml) and [Cu(CH_3_CN)_4_]CF_3_SO_3_ (440 mg, 1.2 mmol) added. After 15 min. a slight turbidity in the colourless solution was observed and after 1.5 h all volatiles were removed *in vacuo* affording a yellowish oil. Trituration with diethyl ether (*ca* 20 ml) and twice with *ca* 20 ml toluene caused the oil to solidify furnishing the colourless microcrystalline product in quantitative yield. A suitable crystal for X-ray diffraction was grown from thf layered with pentane.

NMR (CD_3_CN): ^1^H (300 MHz): δ 2.31 p.p.m. (d, ^3^*J*_PH_ 12.1 Hz, ^1^*J*_CH_ 155 Hz); ^13^C{^1^H} (75 MHz): δ 14.2 p.p.m. (d, ^2^*J*_PC_ 10.1 Hz); ^31^P{^1^H} (121 MHz): δ 142.1 p.p.m. (s).

IR (cm^-1^): 2999 (CH_3_), 2924 (CH_3_), 1605, 1421 (CH_3_), 1284 (CF_3_SO_3_), 1120 (CF_3_SO_3_), 1169 (CF_3_SO_3_), 1049, 1019, 962, 764, 734, 668, 626 and 577.

FAB-MS in nitrobenzyl alcohol matrix (m/*z*): 385 (10%, *M*+H), 401 (15).

### Refinement

All H atoms were positioned geometrically (C—H = 0.98 Å) and constrained to ride on their parent atoms; *U*_iso_(H) values were set at 1.5 times *U*_eq_(C).

### Crystal data

**Table:**

[Cu_2_(CF_3_SO_3_)_2_(C_3_H_9_PS_3_)_2_]	*F*(000) = 768
*M**_r_* = 769.78	*D*_x_ = 1.982 Mg m^−^^3^
Monoclinic, *P*2_1_/*c*	Melting point: 413 K
Hall symbol: -P 2ybc	Mo *K*α radiation, λ = 0.71073 Å
*a* = 8.8347 (15) Å	Cell parameters from 4358 reflections
*b* = 18.306 (3) Å	θ = 2.4–26.3°
*c* = 8.1731 (14) Å	µ = 2.49 mm^−^^1^
β = 102.674 (3)°	*T* = 100 K
*V* = 1289.6 (4) Å^3^	Block, colourless
*Z* = 2	0.10 × 0.08 × 0.04 mm

### Data collection

**Table:**

Bruker APEX CCD area-detector diffractometer	2612 independent reflections
Radiation source: fine-focus sealed tube	2425 reflections with *I* > 2σ(*I*)
graphite	*R*_int_ = 0.021
ω scans	θ_max_ = 26.4°, θ_min_ = 2.2°
Absorption correction: multi-scan (*SADABS*; Bruker, 2002)	*h* = −11→11
*T*_min_ = 0.789, *T*_max_ = 0.907	*k* = −14→22
7349 measured reflections	*l* = −9→10

### Refinement

**Table:**

Refinement on *F*^2^	Primary atom site location: structure-invariant direct methods
Least-squares matrix: full	Secondary atom site location: difference Fourier map
*R*[*F*^2^ > 2σ(*F*^2^)] = 0.030	Hydrogen site location: inferred from neighbouring sites
*wR*(*F*^2^) = 0.075	H-atom parameters constrained
*S* = 1.04	*w* = 1/[σ^2^(*F*_o_^2^) + (0.0393*P*)^2^ + 1.5175*P*] where *P* = (*F*_o_^2^ + 2*F*_c_^2^)/3
2612 reflections	(Δ/σ)_max_ < 0.001
148 parameters	Δρ_max_ = 0.87 e Å^−^^3^
0 restraints	Δρ_min_ = −0.42 e Å^−^^3^

### Special details

**Table:**

Geometry. All e.s.d.'s (except the e.s.d. in the dihedral angle between two l.s. planes) are estimated using the full covariance matrix. The cell e.s.d.'s are taken into account individually in the estimation of e.s.d.'s in distances, angles and torsion angles; correlations between e.s.d.'s in cell parameters are only used when they are defined by crystal symmetry. An approximate (isotropic) treatment of cell e.s.d.'s is used for estimating e.s.d.'s involving l.s. planes.
Refinement. Refinement of *F*^2^ against ALL reflections. The weighted *R*-factor *wR* and goodness of fit *S* are based on *F*^2^, conventional *R*-factors *R* are based on *F*, with *F* set to zero for negative *F*^2^. The threshold expression of *F*^2^ > 2σ(*F*^2^) is used only for calculating *R*-factors(gt) *etc*. and is not relevant to the choice of reflections for refinement. *R*-factors based on *F*^2^ are statistically about twice as large as those based on *F*, and *R*- factors based on ALL data will be even larger.

### Fractional atomic coordinates and isotropic or equivalent isotropic displacement parameters (Å^2^)

**Table:**

	*x*	*y*	*z*	*U*_iso_*/*U*_eq_	
Cu1	0.20918 (3)	0.512402 (17)	0.47201 (4)	0.01851 (11)	
P1	0.02869 (7)	0.58055 (3)	0.31859 (8)	0.01703 (15)	
S1	−0.19119 (7)	0.52875 (3)	0.26779 (8)	0.01850 (14)	
S2	0.03767 (8)	0.61564 (4)	0.07703 (8)	0.02558 (16)	
S3	0.00504 (8)	0.68172 (3)	0.42847 (8)	0.02322 (16)	
S4	0.54023 (7)	0.58664 (3)	0.66022 (8)	0.01807 (15)	
F1	0.66632 (19)	0.70162 (9)	0.8235 (2)	0.0303 (4)	
F2	0.5529 (3)	0.72252 (11)	0.5687 (2)	0.0546 (6)	
F3	0.4170 (2)	0.70427 (11)	0.7523 (3)	0.0495 (5)	
O1	0.4142 (2)	0.57889 (10)	0.5143 (2)	0.0236 (4)	
O2	0.6913 (2)	0.57583 (11)	0.6216 (3)	0.0357 (5)	
O3	0.5166 (2)	0.55310 (12)	0.8091 (2)	0.0346 (5)	
C1	−0.3285 (3)	0.60237 (15)	0.2002 (4)	0.0255 (6)	
H1A	−0.3181	0.6390	0.2895	0.038*	
H1B	−0.4343	0.5827	0.1756	0.038*	
H1C	−0.3073	0.6252	0.0991	0.038*	
C2	0.2468 (3)	0.6205 (2)	0.1033 (4)	0.0350 (7)	
H2A	0.2907	0.6501	0.2024	0.052*	
H2B	0.2720	0.6430	0.0038	0.052*	
H2C	0.2906	0.5712	0.1180	0.052*	
C3	0.0397 (3)	0.65795 (15)	0.6490 (3)	0.0260 (6)	
H3A	−0.0436	0.6259	0.6681	0.039*	
H3B	0.0420	0.7025	0.7159	0.039*	
H3C	0.1394	0.6326	0.6821	0.039*	
C4	0.5454 (3)	0.68447 (15)	0.7022 (4)	0.0253 (6)	

### Atomic displacement parameters (Å^2^)

**Table:**

	*U*^11^	*U*^22^	*U*^33^	*U*^12^	*U*^13^	*U*^23^
Cu1	0.01952 (17)	0.01793 (18)	0.01786 (18)	0.00236 (11)	0.00363 (12)	0.00126 (12)
S1	0.0200 (3)	0.0165 (3)	0.0185 (3)	0.0003 (2)	0.0033 (2)	0.0018 (2)
S2	0.0240 (3)	0.0364 (4)	0.0163 (3)	−0.0004 (3)	0.0043 (3)	0.0077 (3)
S3	0.0312 (3)	0.0145 (3)	0.0231 (3)	0.0005 (3)	0.0041 (3)	0.0012 (2)
S4	0.0199 (3)	0.0153 (3)	0.0187 (3)	−0.0015 (2)	0.0036 (2)	−0.0016 (2)
P1	0.0196 (3)	0.0170 (3)	0.0146 (3)	0.0008 (2)	0.0039 (2)	0.0022 (2)
F1	0.0341 (9)	0.0235 (8)	0.0320 (9)	−0.0079 (7)	0.0045 (7)	−0.0098 (7)
F2	0.0967 (17)	0.0278 (10)	0.0348 (11)	−0.0160 (10)	0.0046 (11)	0.0099 (8)
F3	0.0342 (10)	0.0369 (11)	0.0778 (15)	0.0048 (8)	0.0130 (10)	−0.0267 (11)
O1	0.0255 (10)	0.0248 (10)	0.0189 (9)	−0.0059 (8)	0.0015 (7)	−0.0002 (7)
O2	0.0234 (10)	0.0304 (11)	0.0537 (14)	−0.0018 (8)	0.0095 (9)	−0.0214 (10)
O3	0.0457 (13)	0.0321 (11)	0.0218 (10)	−0.0161 (10)	−0.0014 (9)	0.0064 (9)
C1	0.0225 (13)	0.0222 (14)	0.0294 (15)	0.0042 (10)	0.0002 (11)	0.0080 (11)
C2	0.0248 (14)	0.056 (2)	0.0254 (15)	−0.0064 (14)	0.0072 (11)	0.0069 (14)
C3	0.0373 (15)	0.0226 (14)	0.0176 (13)	0.0004 (11)	0.0053 (11)	−0.0044 (11)
C4	0.0316 (14)	0.0176 (13)	0.0256 (14)	0.0007 (11)	0.0039 (11)	−0.0006 (11)

### Geometric parameters (Å, °)

**Table:**

Cu1—S1^i^	2.2943 (8)	S4—O3	1.419 (2)
Cu1—P1	2.1895 (7)	F1—C4	1.326 (3)
Cu1—O1	2.1466 (18)	F2—C4	1.308 (3)
Cu1—O2^ii^	2.065 (2)	F3—C4	1.338 (3)
P1—S1	2.1192 (9)	C1—H1A	0.9800
P1—S2	2.0941 (9)	C1—H1B	0.9800
P1—S3	2.0886 (10)	C1—H1C	0.9800
S1—C1	1.816 (3)	C2—H2A	0.9800
S2—C2	1.815 (3)	C2—H2B	0.9800
S3—C3	1.814 (3)	C2—H2C	0.9800
S4—C4	1.822 (3)	C3—H3A	0.9800
S4—O1	1.4490 (19)	C3—H3B	0.9800
S4—O2	1.451 (2)	C3—H3C	0.9800
			
P1—Cu1—S1^i^	121.88 (3)	F1—C4—S4	110.88 (19)
P1—S1—Cu1^i^	102.25 (3)	F2—C4—S4	111.7 (2)
S4—O1—Cu1	131.05 (11)	F3—C4—S4	109.64 (19)
S4—O2—Cu1^ii^	132.38 (13)	F1—C4—F3	107.7 (2)
O1—Cu1—S1^i^	105.35 (5)	F2—C4—F1	108.5 (2)
O1—Cu1—P1	104.59 (6)	F2—C4—F3	108.2 (2)
O2^ii^—Cu1—S1^i^	102.03 (7)	S1—C1—H1A	109.5
O2^ii^—Cu1—P1	123.32 (7)	S1—C1—H1B	109.5
O2^ii^—Cu1—O1	95.18 (8)	S1—C1—H1C	109.5
C1—S1—Cu1^i^	110.36 (10)	H1A—C1—H1B	109.5
S1—P1—Cu1	112.23 (4)	H1A—C1—H1C	109.5
S2—P1—Cu1	122.77 (4)	H1B—C1—H1C	109.5
S3—P1—Cu1	112.83 (4)	S2—C2—H2A	109.5
S2—P1—S1	100.09 (4)	S2—C2—H2B	109.5
S3—P1—S1	107.89 (4)	S2—C2—H2C	109.5
S3—P1—S2	99.29 (4)	H2A—C2—H2B	109.5
C1—S1—P1	104.48 (9)	H2A—C2—H2C	109.5
C2—S2—P1	98.73 (10)	H2B—C2—H2C	109.5
C3—S3—P1	101.75 (9)	S3—C3—H3A	109.5
O1—S4—O2	112.64 (13)	S3—C3—H3B	109.5
O3—S4—O1	115.63 (12)	S3—C3—H3C	109.5
O3—S4—O2	116.31 (14)	H3A—C3—H3B	109.5
O1—S4—C4	103.54 (12)	H3A—C3—H3C	109.5
O2—S4—C4	100.87 (13)	H3B—C3—H3C	109.5
O3—S4—C4	105.44 (13)		
			
Cu1—P1—S1—Cu1^i^	47.73 (4)	O3—S4—O1—Cu1	−10.1 (2)
Cu1—P1—S1—C1	162.81 (10)	O3—S4—O2—Cu1^ii^	75.6 (2)
Cu1—P1—S2—C2	−30.47 (13)	C4—S4—O1—Cu1	−124.88 (16)
Cu1—P1—S3—C3	−36.26 (11)	C4—S4—O2—Cu1^ii^	−170.98 (18)
S1^i^—Cu1—P1—S1	−58.38 (5)	S1—P1—S2—C2	−155.34 (12)
S1^i^—Cu1—P1—S2	−177.61 (4)	S1—P1—S3—C3	88.30 (10)
S1^i^—Cu1—P1—S3	63.76 (5)	S2—P1—S1—C1	−65.38 (11)
S1^i^—Cu1—O1—S4	13.26 (16)	S2—P1—S3—C3	−167.85 (10)
P1—Cu1—O1—S4	142.85 (14)	S3—P1—S1—C1	37.90 (11)
S2—P1—S1—Cu1^i^	179.54 (3)	S3—P1—S2—C2	94.47 (12)
S3—P1—S1—Cu1^i^	−77.18 (4)	O1—S4—C4—F1	−173.61 (18)
O1—Cu1—P1—S1	−177.33 (6)	O1—S4—C4—F2	−52.4 (2)
O1—Cu1—P1—S2	63.44 (7)	O1—S4—C4—F3	67.5 (2)
O1—Cu1—P1—S3	−55.19 (6)	O2—S4—C4—F1	−56.9 (2)
O2^ii^—Cu1—P1—S1	76.24 (8)	O2—S4—C4—F2	64.3 (2)
O2^ii^—Cu1—P1—S2	−42.98 (9)	O2—S4—C4—F3	−175.7 (2)
O2^ii^—Cu1—P1—S3	−161.62 (8)	O3—S4—C4—F1	64.5 (2)
O2^ii^—Cu1—O1—S4	−90.74 (16)	O3—S4—C4—F2	−174.3 (2)
O1—S4—O2—Cu1^ii^	−61.2 (2)	O3—S4—C4—F3	−54.3 (2)
O2—S4—O1—Cu1	127.02 (15)		

Symmetry codes: (i) −*x*, −*y*+1, −*z*+1; (ii) −*x*+1, −*y*+1, −*z*+1.
